# Clinical characteristics and prognosis of serous body cavity effusions in patients with sepsis: a retrospective observational study

**DOI:** 10.1186/s12871-018-0621-6

**Published:** 2018-11-14

**Authors:** Ling-Yu Xing, Jun Yin, Mian Shao, Yi-Lin Yang, Ke-Yong Li, Ming-Ming Xue, Su-Cheng Mu, Zhan Sun, Ya-Ping Zhang, Chen-Ling Yao, Xun Chu, Chao-Yang Tong, Zhen-Ju Song

**Affiliations:** 1 Department of Emergency Medicine, Zhongshan Hospital, Fudan University, 180 Fenglin Road, Shanghai, 200032 People’s Republic of China; 2 Department of Pharmacology, University of Virginia School of Medicine Charlottesville, Virginia, 22908 USA; 3 Xinhua Hospital, Shanghai Institute for Pediatric Research, Shanghai Jiao Tong University School of Medicine, 1665 Kongjiang Road, Shanghai, 200092 People’s Republic of China

**Keywords:** Sepsis, Serous body cavity effusions, Bloody effusions, VEGFR2, Ang2, E-selectin, sVCAM-1, Inflammatory mediators

## Abstract

**Background:**

Cavity effusion is common in patients with infectious diseases. However, the incidence rate and characteristics of serous cavity effusions (SCE) in septic patients are not clear to date. The objective of this study was to investigate the incidence and characteristics of SCE in septic patients and to explore the correlations between the bloody effusions and the illness severity/prognosis in septic patients.

**Methods:**

From January 2010 to January 2015, a total of 214 patients with severe sepsis and septic shock were enrolled in this retrospective observational study. Thoracentesis or abdominal paracentesis was performed in 45 septic patients because of massive pleural effusions or ascites. The serum concentrations of VEGF, VEGFR, Ang, sICAM-1, sVCAM-1, E-selectin, Serpine1 and VE-cadherin in 45 septic patients underwent paracentesis were measured by enzyme-linked immunosorbent assay (ELISA).

**Results:**

Of the 214 septic patients, 155 (72.4%) had SCE according to imaging or ultrasound manifestations. 45 subjects with SCE underwent therapeutic thoracentesis or abdominal paracentesis. Effusion laboratory analysis showed that exudates were predominant when compared with transudates (95.6% vs. 4.4%), and 16 (35.6%) patients suffered bloody effusions. Compared with patients with non-bloody effusions, those with bloody effusions showed higher critical illness scores (13 vs. 17 for APACHE II; 7 vs. 9 for SOFA), and higher mortality (6.9% vs. 62.5%). Moreover, patients with bloody effusions had delayed TT and APTT, increased D-dimer concentration, and higher serum levels of CRP and PCT (*P* < 0.05). In addition, the serum levels of Ang2, sVCAM-1 and E-selectin were significantly higher in patients with bloody effusions than in those with non-bloody effusions (*P* < 0.05). However, the serum level of VEGFR2 was lower in patients with bloody fluids (*P* = 0.025).

**Conclusions:**

The incidence of serous cavity effusion is high in patients with sepsis. The septic patients with bloody effusions suffer a more inflammatory burden and a worse prognosis compared to septic patients with non-bloody effusions.

## Background

Severe sepsis and septic shock are major causes of noncardiac death in critically ill patients [1]. Despite progress in the development of antibiotics and other supportive care therapies, severe sepsis remains an unconquered challenge for clinicians with an unacceptable high mortality rate of 30 to 50% [2]. In view of its high morbidity, mortality and care costs, clinicians are encountering a great challenge in diagnosing and treating the disease.

In clinical observation, we found that serous body cavity effusions (SCE) are common in most patients with severe sepsis, and bloody effusions are associated with a higher mortality. The clinical significance and pathogenesis of body cavity effusions have been investigated in different kinds of inflammatory conditions (such as tuberculosis, other bacterial and viral infections) and trauma. However, the incidence, prognosis and clinical characteristics of SCE in sepsis patients are rarely studied to date.

Although the exact pathogenesis of SCE in infectious diseases, especially sepsis, is unclear, there are several mechanisms might be responsible for the formation of SCE. For example, hypoproteinemia secondary to serious bacterial, fungal, and viral infections may lead to the alteration of plasma osmotic pressure. Additionally, systemic capillary leakage due to the inflammatory response and the apoptosis of endothelial cells play a more important role in pathophysiology of sepsis and the formation of effusions. It is well known that sepsis is a systemic inflammatory response syndrome caused by infection. During the systemic inflammatory response, plenty of inflammatory mediators and adhesion molecules, such as tumor necrosis, interleukins, soluble intercellular adhesion molecules (sICAM) and soluble vascular cell adhesion molecules (sVCAM), are released in cascade to injure the vascular endothelium during sepsis [3, 4]. Vascular endothelial growth factor (VEGF) and the ligand family of tyrosine kinases, including angiopoietin-1, 2 (Ang-1, Ang-2) have a potent angiogenic effect but lead to an increased permeability of new capillaries [5, 6]. Additionally, the contraction of endothelial cells, cytoskeleton remodeling, and the increased pinocytosis of endothelial cells have been demonstrated to increase vascular permeability during the inflammatory reaction. Thus, dysfunction of the endothelium barrier increases vascular permeability and microvascular leakage [7].

Given few reports to explore the incidence, characteristics and pathogenesis of body cavity effusions occurring during the clinical course of sepsis, we designed this retrospective observational study to investigate the incidence and characteristics of SCE in septic patients, and to explore the correlations between the bloody effusions and the illness severity/prognosis in septic patients.

## Methods

### Study design and enrollment

**Fig. 1:**
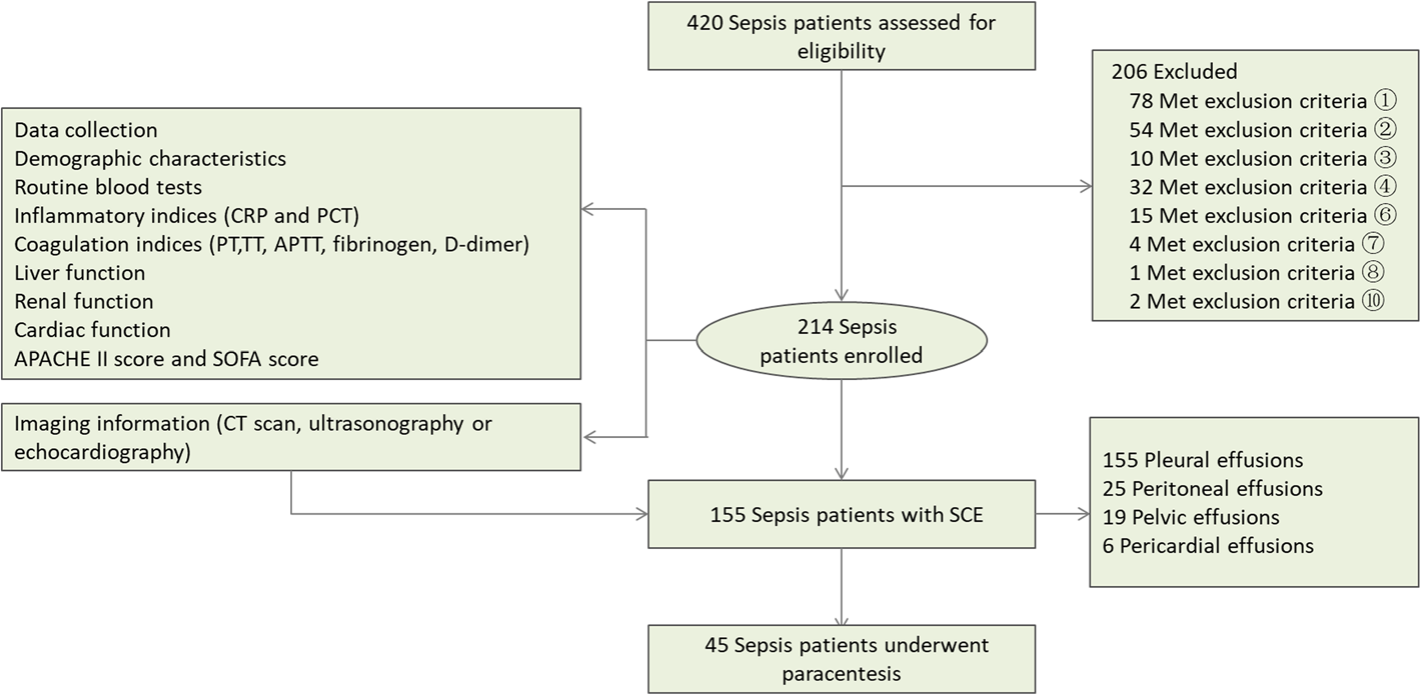
The flowchart to illustrate the recruit of study samples

From January 2010, all consecutive intensive care unit (ICU) patients admitted to our Emergency ICU of Zhongshan Hospital, Fudan University for management of sepsis, were prospectively included in a Sepsis Database. From January 2010 to January 2015, a total of 214 patients with severe sepsis and septic shock enrolled in this study were selected from this database. All sepsis patients were enrolled in strict accordance with the international guidelines for the management of severe sepsis and septic shock [8]. Patients were excluded if they had one of the following: ①chronic heart failure (NYHAIII-IV) [9]; ②chronic renal insufficiency; ③chronic hepatic dysfunction (Child-Pugh scores > 10) [10]; ④hypoproteinemia (< 30 g/L) due to chronic malnutrition; ⑤patients with congenital or acquired hematological diseases; ⑥malignant tumors; ⑦bloody effusion caused by trauma or iatrogenic performance (including pericardicentesis, thoracentesis and abdominal paracentesis); ⑧patients less than 18 years old; ⑨pregnant women; ⑩patients who died of pericardial tamponade, especially bloody pericardial tamponade. Pericardial tamponade can lead to acute circulatory failure and cardiac arrest directly, making the bloody effusions the cause of death rather than sepsis. Therefore, we exclude this kind of patients in our study. A flowchart to illustrate the recruit of study samples was shown in Fig. 1. The study was approved by the ethics committee of Zhongshan Hospital of Fudan University (Shanghai, China) (record number 2006–23). Written informed consent was obtained from subjects or from their legal surrogates before enrollment.​

### Data collection

Medical charts were retrospectively reviewed. Firstly, demographic characteristics and clinical data were recorded: demographic data, routine blood tests, inflammatory indices (CRP and PCT), coagulation indices (prothrombin time (PT), thrombin time (TT), activated partial thromboplastin time (APTT), fibrinogen and D-dimer), blood biochemistry indices including hepatic, renal and cardiac function (bilirubin, liver enzymes, albumin, creatinine, urea nitrogen, creatine kinase and N-terminal probrainnatriuretic peptide (NT-proBNP)), Acute Physiology and Chronic Health Evaluation (APACHE) II score, Sequential Organ Failure Assessment (SOFA) score on admission, and the 28-day mortality rate. Moreover, we also collected imaging information, including the CT scan of chest, abdomen and pelvis, ultrasonography or echocardiography, which was necessary to determine if body cavity effusions existed.

In this study, a total of 45 septic patients underwent diagnostic or therapeutic puncture or drainage with the following symptoms: moderate to massive effusions leading to organ oppression (such as respiratory failure and tamponade); continuous fever or deterioration of organ function after treatment. The attending doctors performed the paracentesis under the guidance of bedside ultrasound. And then, the laboratory analysis of serous cavity fluids was performed. The following characteristics of effusions were tested and recorded: color, specific gravity, WBC, RBC, lactate dehydrogenase (LDH), glucose and protein levels. Effusion was classified either as exudate or transudate by Lights criteria, and either as bloody or non-bloody fluid based on the test results [11, 12].

### Measurement of VEGF, VEGFR1, VEGFR2, Ang1, Ang2, sICAM-1, sVCAM-1, VE-cadherin, E-selectin and Serpine1 levels

Blood specimens of 45 septic patients who underwent puncture as detailed above were collected after a definitive diagnosis of severe sepsis and septic shock. The blood samples were collected in plain sterile tubes, centrifuged at 3000 rpm at 4 °C for 10 min, and then the supernatant was stored at − 80 °C for use. The levels of VEGF, VEGFR1, VEGFR2, Ang1, Ang2, sICAM-1, sVCAM-1, VE-cadherin, E-selectin and Serpine1 were measured by enzyme-linked immunosorbent assay method (R & D Systems, Minnesota, USA) according to the manufacturer’s instructions.

### Statistical analysis

Shapiro-Wilk test was used for testing normality. Normally distributed continuous variables were expressed as means ± standard deviations, abnormally distributed continuous variables were expressed as median (the 25th and 75th quartiles). Student’s t test or one-way analysis of variance was used to compare distribution continuous variables. Kruskal-Wallis one-way analysis or Mann-Whitney U test was utilized to compare abnormal distribution continuous variables. Categorical data were expressed as number (percentage) and compared with Pearson’s chi-square test or Fisher exact test when appreciate. The differences of serum concentrations of VEGF, VEGFR, Ang, sICAM-1, sVCAM-1, E-selectin, Serpine1 and VE-cadherin between patients with bloody effusion and non-bloody effusion groups were compared using multivariate logistic regression adjusting for potential confounding variables including APACHE II and SOFA scores. All statistical analyses were two-sided, and the significance level was set to *P* < 0.05. The software package SPSS, version 19.0 (SPSS Inc., Chicago, IL, USA) was used for data analysis.

Sample size (*n* = 45) was calculated by the standard deviation of the first 20 patients. An error of 0.05 was set up and powers of 4 main variables (E-selectin, SVCAM1, Ang2 and VEGFR2) were calculated based on the combined standard errors. They were 89.6%, 99.9%, 80.1% and 87.1% respectively. The software package SAS 9.4 was used for power study calculation.

## Results

### The characteristics and the identification of SCE in septic patients

From January 2010 to January 2015, a total of 214 septic patients, including 134 males and 80 females, were enrolled in this retrospective study. The mean age of septic patients was 62.3 ± 15.4 years. The overall 28-day mortality rate of patients with severe sepsis and septic shock was 36.9%. The primary sources of infection were the lungs (67.3%), followed by abdomen (24.8%), blood stream (0.8%), urinary tract (0.8%), and others (6.5%). The baseline characteristics and laboratory data of these patients were shown in Table 1. According to the CT scan, ultrasonography and echocardiography 155 patients (72.4%) had SCE. The locations of the effusions were pleural (105 cases), peritoneal (25 cases), pelvic (19 cases) and pericardial (6 cases). The multiple SCE were used to indicate the presence of at least two cavity effusions, and 73 (47.1%) cases met this criterion (Table 1).

**Table 1:** Clinical characteristic of 214 patients with sepsis

Variables	Number (%) or Median (25–75%)
Total patients	214
Male	134 (62.6%)
Survival	135 (63.1%)
Age (y)	62.3 ± 15.4
Source of sepsis	
Lung	144 (67.3%)
Abdominal	53 (24.8%)
Urinary tract	2 (0.8%)
Bloodstream	2 (0.8%)
Others	14 (6.5%)
APACHE II score	15 (12–20)
SOFA score	8 (7–10)
MODS	
Liver failure	16 (7.5%)
Renal failure	42 (19.6%)
Septic shock	50 (23.4%)
ARDS	76 (35.5%)
DIC	8 (3.7%)
RBC (× 10^12^/L)	4.15 (3.23–5.76)
Hg (g/L)	125.0 (116.6–135.8)
PLT (× 10^9^/L)	173.8 (121.3–205.7)
PT (s)	16.7 (13.2–20.8)
TT (s)	17.8 (13.4–21.6)
APTT (s)	35.7 (33.4–40.8)
Fibrinogen (mg/dL)	412.2 (383.2–520.3)
D-dimer (mg/L)	9.6 (8.2–14.6)
Serum Albumin (g/L)	29.4 (23.4–35.9)
CRP (mg/L)	117.5 (93.6–230.4)
PCT (ng/mL)	15.3 (11.4–26.7)
WBC (×10^9^/L)	13.4 (8.2–21.3)
Neutrophil (%)	0.84 (0.62–0.88)
Body cavity effusion	155 (72.4%)
Pleural effusion	105 (67.7%)
Peritoneal effusion	25 (16.1%)
Pelvic effusion	19 (12.3%)
Pericardial effusion	6 (3.9%)
Multiple body cavity effusion	73 (47.1%)
Pleural + Peritoneal	20 (27.4%)
Pleural + Pelvic	17 (23.3%)
Pleural + Pericardial	4 (5.5%)
Peritoneal + Pelvic	16 (21.9%)
Pleural + Peritoneal + Pelvic	16 (21.9%)

Note: Data are expressed as numbers (%), means ± standard deviations, or medians (the 25th and 75th quartiles)

### The effusions properties of septic patients with SCE

A total of 45 septic patients with SCE underwent diagnostic or therapeutic thoracentesis (*n* = 30) and abdominal paracentesis (*n* = 15). Effusion laboratory analysis showed that exudate (*n* = 43, 95.6%) was more common than transudate (*n* = 2, 4.4%), and 16 (35.6%) patients had bloody effusion (Table 2). The mortality rate of patients with bloody effusions was 62.5%, which was significantly higher than those with non-bloody effusions (6.9%) (*P* = 0.012, OR = 15.33, 95% CI 1.92–122.82). The APACHE II and SOFA scores in patients with bloody effusions were significantly higher than those in patients with non-bloody effusions (17 vs. 13, *P* = 0.032 for APACHE II, 9 vs. 7, *P* = 0.04 for SOFA, respectively). No significant difference was in age, gender ratio, infection sites and complications between blood effusion and non-bloody effusion groups.

**Table 2:** The characteristics of patients without effusion, with bloody and non-bloody effusion

Variables	Non-bloody effusion	Bloody effusion	Without effusion	*P*_1_	*P*_2_
Total number	29	16	59		
Age (y)	61.7 ± 14.6	63.4 ± 13.9	59.7 ± 14.2	0.18	0.24
Male	22 (75.9%)	11(68.7%)	36 (61.0%)	0.42	0.73
28-d mortality	2 (6.9%)	10 (62.5%)	6 (10.1%)	6.94 × 10^−6^	0.012
Infective sites					
Lung	11	7	30	0.51	0.25
Abdominal	15	8	27	0.92	0.91
Others	3	1	2	0.53	1.00
APACHE II	13 (11–17)	17 (12–19)	11 (9–15)	0.011	0.032
SOFA	7 (6–10)	9 (8–12)	3 (2–5)	0.0091	0.042
Organ failure					
Liver failure	4	5	3	0.12	0.25
Renal failure	8	7	1	3.72 × 10^−5^	0.33
Septic shock	7	3	1	0.0029	0.73
ALI/ARDS	7	5	1	0.0011	0.73
DIC	2	1	1	0.36	1.00
Laboratory indices					
RBC (×10^12^/L)	4.2 (3.6–7.8)	3.8 (3.6–7.9)	4.3 (3.6–4.7)	0.12	0.14
Hg (g/L)	123.8 (116.2–143.2)	114.8 (109.6–121.7)	131.2 (107.1–145.6)	0.18	0.21
PLT (×10^12^/L)	169.0 (126–180)	148.0 (113–167)	140.0 (96–230)	0.22	0.24
PT (s)	16.0 (8–18)	18.0 (9–21)	13.0 (12–16)	0.09	0.15
TT (s)	16.8 (9.2–19.3)	22.4 (12.1–25.2)	16.8 (14.8–17.9)	0.013	0.012
APTT (s)	37.6 (29.4–43.2)	47.5 (36.8–56.6)	31.8 (27.9–38.7)	0.0078	0.011
Fibrinogen (mg/dL)	426.6 (362.5–510.2)	369.4 (342.2–496.5)	391.0 (306.5–494.7)	0.022	0.020
D-dimer (mg/L)	7.2 (6.3–10.8)	13.0 (8.45–11.2)	4.5 (1.8–11.5)	0.0010	0.0039
Serum Albumin (g/L)	28 (24–32)	28 (26–31)	29 (25–36)	0.83	0.87
CRP (mg/L)	100.9 (72.5–156.8)	131.2 (95.5–210.8)	84.7 (45.8–97.2)	6.79 × 10^−5^	0.021
PCT (ng/mL)	18.2 (4.78–20.5)	22.6 (6.42–31.5)	2.25 (0.3–19.4)	5.89 × 10^− 5^	0.031
Analysis of effusions					
Proportion	1.02 (1.02–1.03)	1.02 (1.02–1.03)			0.86
WBC number/mL	1517 (1340–2650)	2581 (2240–3415)			0.028
RBC number/mL	1424 (1252–2145)	18,916 (15671–57,824)			0.0001
LDH	716.0 (654–1055)	1867 (892–2162)			0.0031
Albumen	19.5 (18.5–27.6)	27.0 (25.5–32.5)			0.021
Glucose	8.5 (8.0–10.5)	9.2 (7.5–11.4)			0.16

Note: Data are expressed as numbers (%), means ± standard deviations, or medians (the 25th and 75th quartiles). *P*_*1*_, differences among septic patients without SCE, with bloody effusion and non-bloody effusion groups; *P*_*2*_, differences between patients with bloody effusion and non-bloody effusion groups

The serum concentrations of C-reactive protein and PCT in three groups (patients without SCE, patients with non-bloody effusions and patients with bloody effusions) were shown in Table 2. Our results showed that there were significant differences among the three groups (*P* = 0.0007 for CRP, *P* = 0.0006 for PCT, respectively). The serum levels of CRP and PCT in patients with bloody (131.2 mg/L and 22.6 ng/mL) and non-bloody effusions (100.9 mg/L and 18.2 ng/mL) were significantly higher than those in patients without effusions (84.7 mg/L, 2.25 ng/ml). Moreover, the differences were significant between bloody effusion and non-bloody effusion groups (*P* = 0.02 for CRP, *P* = 0.03 for PCT, respectively). In addition, there were significant differences among the three groups regarding the coagulation and fibrinolysis indices, including TT, APTT and D-dimer (*P* = 0.013 for TT, *P* = 0.008 for APTT, *P* = 0.001 for D-dimer, respectively). And the levels of those markers in patients with bloody effusions were also higher than those in patients with non-bloody effusions (22.4 s vs. 16.8 s, *P* = 0.012 for TT; 47.5 s vs. 37.6 s, *P* = 0.011 for APTT and 13.0 mg/L vs. 7.2 mg/L, *P* = 0.004 for D-dimer, respectively). Additionally, the routine and biochemistry analysis of effusion revealed higher levels of WBC, RBC, LDH and albumin in patients with bloody effusions (Table 2).

### The levels of VEGF, VEGFR, Ang, and various adhesion molecules between septic patients with bloody and non-bloody effusions

**Fig. 2:**
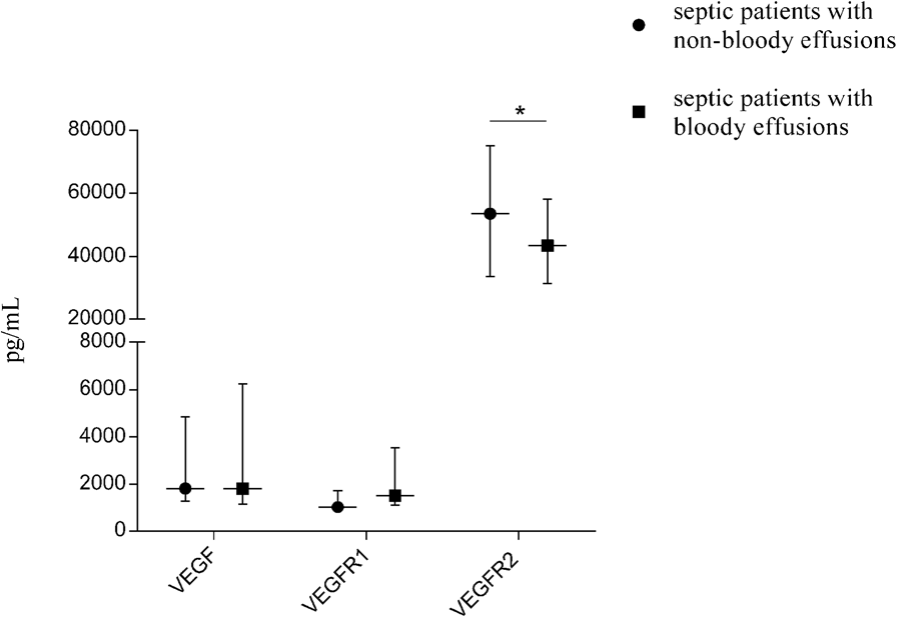
The levels of VEGF and VEGFR in septic patients with or without bloody effusions. The serum levels of VEGF, VEGFR1 and VEGFR2 were tested by ELLISA in non-bloody group and bloody group. There were no significant differences in VEGF and VEGFR1 between the two groups. The VEGFR-2 was significantly decreased in patients with bloody effusions compared to patients with non-bloody effusions (43,510 pg/mL vs. 53,495 pg/mL; *P*_adj_ = 0.025). *P*_adj_ value was calculated using logistic regression adjusted for APACHE II and SOFA scores. *P < 0.05

**Fig. 3:**
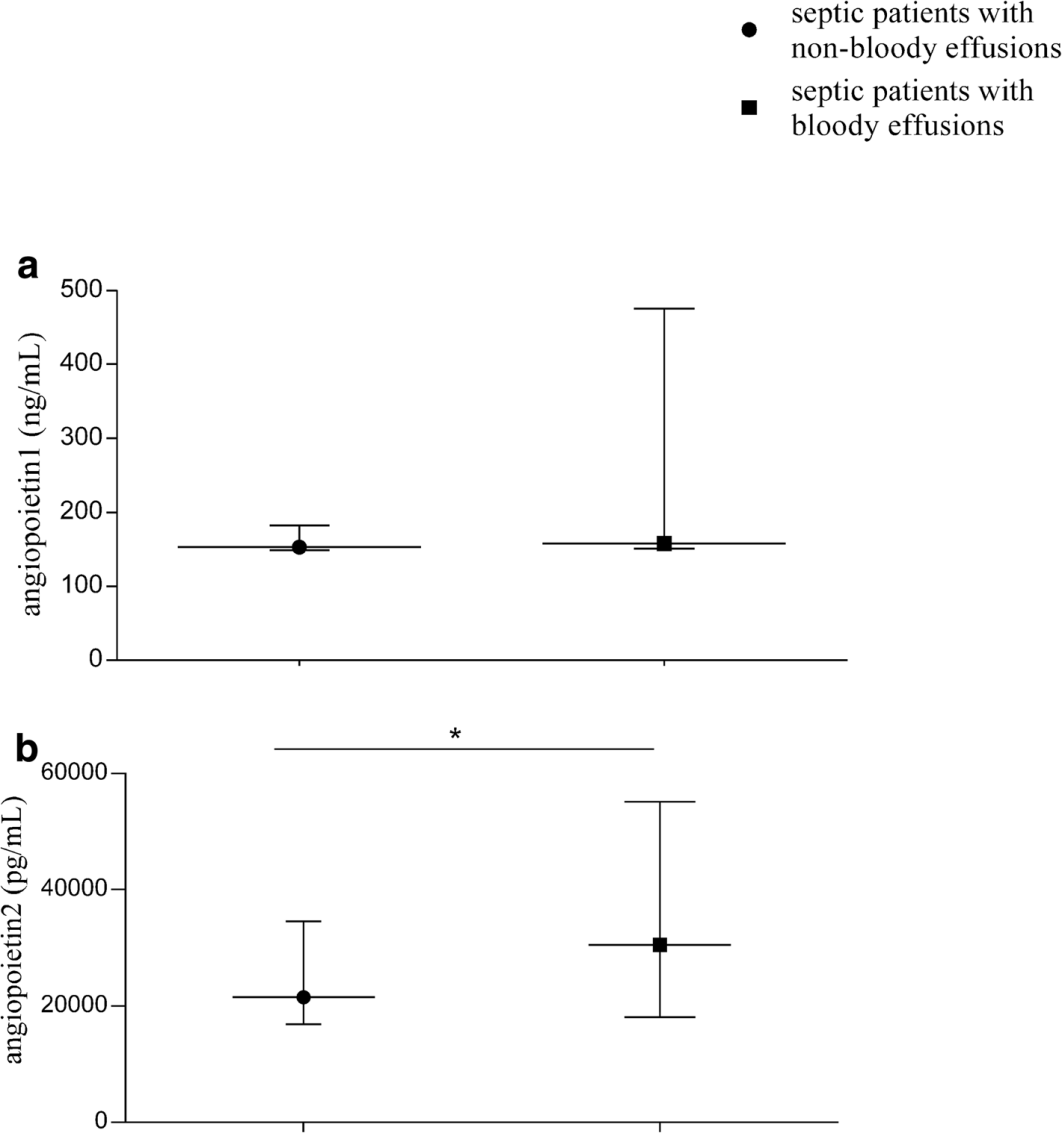
The serum levels of Ang-1 and Ang-2 in septic patients with non-bloody effusions and bloody effusions. We used ELLISA method to test the Ang-1 and Ang-2 in serum. The results showed a) There was no significant difference in Ang-1 between the two groups; b) The level of Ang2 was remarkably increased in bloody effusion group, when compared with the non-bloody effusion group (30,527 pg/mL vs. 21,536 pg/mL; *P*_adj_ = 0.018). *P*_adj_ value was calculated using logistic regression adjusted for APACHE II and SOFA scores. *P < 0.05

**Fig. 4:**
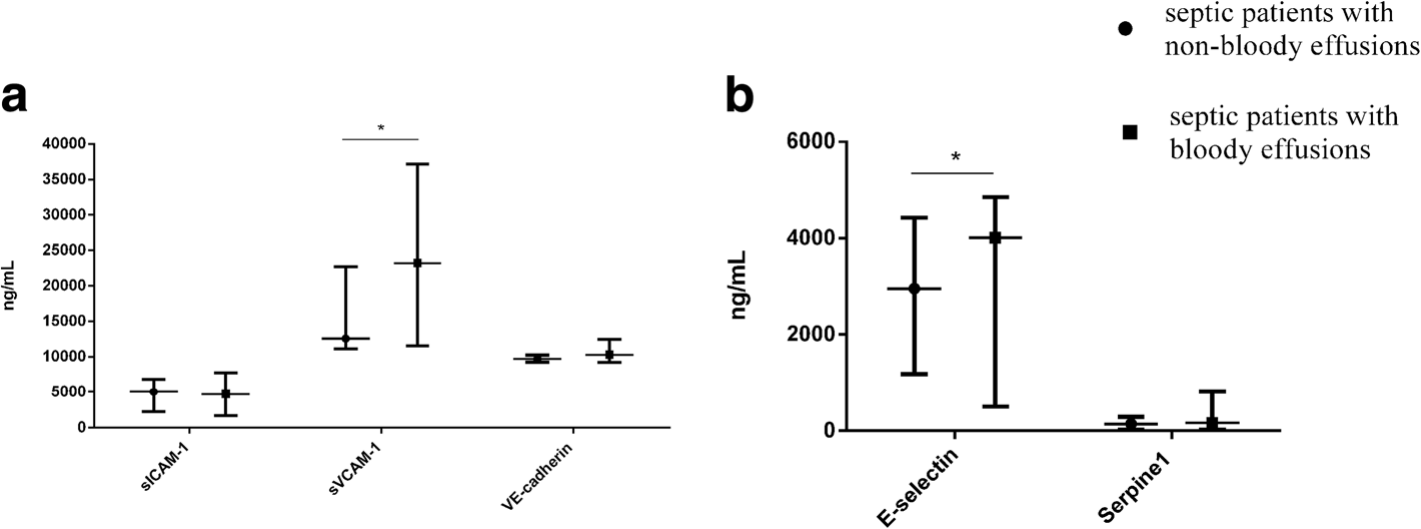
The serum levels of adhesion molecules and E-selectin, Serpine-1 between non-bloody effusions and bloody effusions goups. We used ELLISA method to test the above molecules. The results showed a) The level of sVCAM-1 was significantly higher in bloody effusion patients than in non-bloody effusion patients (23,191 ng/mL vs. 12,545 ng/ml, *P*_adj_ = 0.012); while there were no significant differences in sICAM-1 and VE-cadherin between the two groups; b) The level of E-selectin was dramatically higher in bloody effusion group than in non-bloody group (4014 ng/mL vs. 2951 ng/ml, *P*_adj_ = 0.024). And no difference was found in serpine-1 between the two groups. *P*_adj_ value was calculated using logistic regression adjusted for APACHE II and SOFA scores. *P < 0.05

The serum levels of VEGF and VEGFR (VEGFR1 and VEGFR2) were shown in Fig. 2. The level of VEGFR-2 was significantly decreased in patients with bloody effusions compared to patients with non-bloody effusions (43,510 pg/mL vs. 53,495 pg/mL; *P*_*adj*_ = 0.025). However, the level of Ang2 remarkably increased in bloody effusion group, when compared with the non-bloody effusion group (30,527 pg/mL vs. 21,536 pg/mL; *P*_*adj*_ = 0.018) (Fig. 3). Moreover, the levels of sVCAM-1 and E-selectin were significantly higher in bloody effusion patients than those in non-bloody effusion patients (23,191 ng/mL vs. 12,545 ng/ml, *P*_*adj*_ = 0.012; 4014 ng/mL vs. 2951 ng/ml, *P*_*adj*_ = 0.024, respectively) (Fig. 4).

## Discussion

To the best of our knowledge, this was the first retrospective observational study to describe the frequency and clinical characteristics of serous body cavity effusions in patients with severe sepsis, and to explore the association between the bloody fluids and illness severity/prognosis in septic patients. Our study revealed that the incidence of SCE in severe sepsis patients was up to 72.4%, exudate was more common than transudate, and bloody effusions were associated with a significantly high mortality. Furthermore, we also explored the relationship between bloody effusions and the related clinical events including inflammatory reactions, coagulation and vascular endothelial growth markers. Our results indicate that the patients with bloody effusions suffer a more inflammatory burden, a more imbalance of coagulation/fibrinolysis and dysfunction of endothelium barrier compared to septic patients with non-bloody effusions.

Serous cavity effusions in sepsis might be caused by several factors, which including destruction of vascular endothelial cells triggered by inflammatory cytokines [7], high vascular permeability [4] and the reduction of colloid osmotic pressure. Mechanisms underlying occurrence of pleural effusion have been investigated in cases of bacterial, fungal, and viral infections. Participation of inflammatory cytokines such as interleukin (IL)-6, IL-8, vascular endothelial growth factor and tumor necrosis factor have received considerable attention [13]. The results of our study also support that excessive inflammatory response is associated with the formation of SCE. Both CRP and PCT proteins were produced in the acute phase during inflammation, and can be used as biomarkers to determine inflammation levels [14]. Our findings showed that the levels of these two biomarkers were significantly higher in septic patients with bloody effusions than in patients with non-bloody effusions, suggesting that a much more severe inflammation reaction exists in septic patients with bloody effusions.

VEGF and its receptors are mediators with important functions in angiogenesis (vascular endothelial cell proliferation, differentiation, and tube formation) [15]. They play an important role in body effusion formation by increasing vascular permeability and vascular leakage, in addition to t their involvement in vascular development [16]. Increase of vascular permeability and fluid leakage underlies the mechanism in the formation of body cavity effusion. Several previous studies showed that circulating sVEGFR-2 level was decreased in septic patients [17, 18]. In addition, a clinical observational study reported that sVEGFR-1 level was increased significantly in patients with ARDS, while VEGF level had no change [19]. The resultant impaired VEGFR-2 signaling is likely to hinder endothelial recovery during sepsis. The precise role of VEGFR1 remains unclear, but an abundance of evidence supports a negative regulatory role on VEGF bioactivity, acting as a decoy or silent receptor to sequester VEGF from VEGFR2 binding [20]. In this study, we found that the serum level of VEGFR-2 was significantly decreased in septic patients with bloody effusions compared to patients with non-bloody effusions. While there were no significant differences in VEGF and VEGFR1 level between the two groups. As we know, Angiogenesis is a very complicated process that involves many angiogenic factors, among which angiopoietin (Ang) has been well studied. Evidence indicates that the ligand family of Tie-2 (tyrosine kinase with immunoglobulin-like and EGF homology domains 2), Ang-1 and Ang-2, mediates different functions of angiogenesis [21–23]. Ang-1 and Ang-2 can bind to and inhibit the Tie-2 receptor on endothelial cells. The circulating level of Ang-1 exceeds that of Ang-2 under physiological condition, thus enabling a preferential interaction between Ang-1 and the Tie-2 receptors. However, the inflammatory response in sepsis causes the overproduction of Ang-2, which results in a shift toward an Ang-2-Tie-2 interaction. This interaction will promote pro-inflammatory and pro-thrombotic pathways, microvascular leak and angiogenic stimuli [24]. Previous studies found that elevated Ang-2 levels are correlated with the severity of illness and adverse outcomes in patients with sepsis [25, 26], which is consistent with our findings that the level of Ang-2 is increased significantly in septic patients with bloody effusions than non-bloody effusions. Taken together, all of these findings indicate that VEGFR2 and Ang-2 should be involved in the formation of body cavity effusion, especially bloody effusion.

Growing evidence indicates the surface of endothelial cells is covered with cell adhesion molecules that mediate the adhesion and extravasation of leukocytes and play a pivotal role in causing breakdown of the vascular endothelium, which in turn leads to body cavity effusions [27–29].Among these adhesion molecules, sICAM-1, sVCAM-1 and E-selectin have a significant effect in aberrant leukocyte activation and recruitment into host tissues [4, 30]. sICAM-1 and sVCAM-1 are extensively expressed on activated endothelial cells. It has been reported that sVCAM-1 and sICAM-1 enhance the adhesion of leukocytes and inflammatory cells to endothelial cells, then promote the dysfunction and injury of endothelial cells [31]. In addition, E-selectin is synthesized in endothelial cells, and the serum level of E-selectin increases in response to inflammation and other stimulations and it is usually used as a molecular marker of endothelial injury. The unique function of E-selectin is represented by the direct interaction between leukocytes and the vascular endothelium via binding to the carbohydrate epitopes on leukocytes or endothelial cells [32]. Our results showed that the serum levels of sVCAM-1 and E-selectin were increased in septic patients with bloody effusions than those in septic patients with non-bloody effusions, supporting the notion that the aberrant leukocyte activation and recruitment exert enormous influence on the formation of bloody effusion.

In addition, the disturbance of coagulation/fibrinolysis is involved in the development of severe sepsis [33, 34]. The current findings showed that the coagulation indices (TT, APTT and D-dimer) in patients with bloody effusions were significantly higher than in patients with non-bloody effusions. However, the level of fibrinogen was lower in the bloody effusions group than in the non-bloody group. All these results might demonstrate that the disturbance of coagulation/fibrinolysis was involved in the progression of bloody serous cavity effusions. However, the changes in coagulation and fibrinolysis are complex in sepsis, and D-dimer, APTT and TT are not sensitive enough to reflect them. More sensitive and specific biomarkers are needed to illustrate the relationship between the disturbance of coagulation and formation of serous cavity effusion.

Several limitations of this study must be mentioned. First, it was a single-center retrospective observational study. A potential selection bias might exist because only 30% septic patients with SCE in this study underwent diagnostic or therapeutic thoracentesis. Second, we did not investigate the levels of VEGF, VEGFR, Ang and various adhesion molecules in body cavity effusions, which could provide further clues to the formation of bloody effusion. Third, we neglected the variable gene expression which is patient specific and it might have an effect on serum levels. Last, a control group of ICU patients without SCE should be included to clearly demonstrate the association between acute inflammatory response and the formation of effusion. Therefore, a prospective and large-sample study is needed to confirm the influential factors of the pathogenesis of serous cavity effusion, especially bloody fluids in septic progression.

## Conclusions

In summary, our results demonstrate that exudative effusions are common in severe sepsis patients, and bloody effusion is associated with a significantly higher mortality. Additionally, our findings suggest that the inflammatory response, the disturbance of coagulation/fibrinolysis and dysfunction of endothelium barrier could contribute to the production of bloody fluids in septic progression. Clinicians should be aware of these poor prognostic features.

## Abbreviations

Ang: Angiopoietin
APACHE II: Acute physiology and chronic health evaluation II
APTT: Activated partial thromboplastin time
CI: Confidence interval
CRP: C reactive protein
CT scan: Computed tomography scan
ELISA: Enzyme linked immunosorbent assay
ICU: Intensive care unit
IQR: Interquartile range
LDH: Lactate dehydrogenase
NT-proBNP: N-terminal probrainnatriuretic peptide
NYHA: New York heart function assessment
OR: Odds ratio
PCT: Procalcitonin
PT: Prothrombin time
RBC: Red blood cell
SCE: Serous cavity effusion
sICAM-1: soluble intercellular adhesion molecule-1
SOFA: Sequential organ failure assessment
sVCAM-1: soluble vascular cell adhesion molecule-1
TT: Thrombin time
VE-cadherin: Vascular endothelial cadherin
VEGF: Vascular endothelial growth factor
VEGFR: Vascular endothelial growth factor receptor
WBC: White blood cell
